# Management of Pediatric Facial Burns with Zinc-Hyaluronan Gel

**DOI:** 10.3390/children9070976

**Published:** 2022-06-29

**Authors:** Aba Lőrincz, Anna Gabriella Lamberti, Zsolt Juhász, András Garami, Gergő Józsa

**Affiliations:** 1 Department of Thermophysiology, Institute for Translational Medicine, Medical School, University of Pécs, 12 Szigeti Street, H7624 Pécs, Hungary; aba.lorincz@gmail.com (A.L.); lambi.anna.gabi@gmail.com (A.G.L.); andras.garami@aok.pte.hu (A.G.); 2 Division of Surgery, Traumatology and Otorhinolaryngology, Department of Pediatrics, Clinical Complex, University of Pécs, 7 József Attila Street, H7623 Pécs, Hungary; juhasz.zsolt.dr@pte.hu

**Keywords:** pediatric, facial burn, partial-thickness burn, zinc-hyaluronate

## Abstract

Zinc-hyaluronan-containing burn dressings have been associated with enhanced reepithelialization and low infection rates, although their effectiveness has not yet been investigated in pediatric facial thermal injuries. This single-arm, retrospective cohort study assessed the characteristics of 23 children (≤17-year-old) with facial superficial partial-thickness burns and the wound closure capabilities of the applied zinc-hyaluronan gel. Patients were admitted consecutively to the Pediatric Surgery Division in Pécs, Hungary, between 1 January 2016 and 15 October 2021. The mean age of the children was 6.2 years; 30.4% of them were younger than 1 year. An average of 3% total body surface was injured in the facial region and 47.8% of the patients had other areas damaged as well, most frequently the left upper limb (30.4%). The mean time until complete reepithelialization was 7.9 days and the children spent 2 days in the hospital. Wound cultures revealed normal bacterial growth in all cases and follow-up examinations found no hypertrophic scarring. In conclusion, pediatric facial superficial partial-thickness burns are prevalent during infancy and coincide with left upper limb injuries. Rapid wound closure and low complication rates are accountable for the moderate amount of hospitalization. These benefits, along with the gel’s ease of applicability and spontaneous separation, are linked to child-friendly burn care.

## 1. Introduction

Pediatric burns constitute a great challenge to patients, their families, and healthcare providers because of their high incidence rate (1–7 million/year) and frequent complications (49% of all cases) [1,2]. The facial region is assumed to be injured in 39% of all burns and the most common etiology is scalding [3]. Children, especially infants, have thinner skin—compared to adults—while their immune system and defensive reflexes are still in development [4,5]. Also, their experience with the environment is limited, in addition to their natural curiosity towards it. These factors combined are responsible for the substantial likelihood of severe traumatic and burn injuries in the pediatric population. Facial burns can be responsible for lifelong deformities due to scar formation and restriction of the facial structure’s growth potential. Later, this may lead to severe functional and psychological consequences, such as blindness, as well as eating, mood, and anxiety disorders [6,7].

Thermal injuries can be categorized by their depth. Superficial burns (also called I degree) only involve the epidermis and they appear as painful and red wounds. The papillary stratum is damaged in superficial partial-thickness thermal injuries (alas II/1 degree), which can be characterized by bullae and painful, moist, pink wound beds. Deep partial-thickness burns (II/2 degree) damage the stratum reticulare as well, while the wound bed turns numb and dry with a purple or white color [8]. Conservative treatments, involving dressings (such as silver foam, biosynthetic, amnion membrane dressings, or xenografts) and ointments (for example, silver sulfadiazine, silver nitrate, or hydrogels) are recommended in I and II/1 degree burns [9,10], while surgical reconstruction with various skin grafts, tissue expansion, and flaps are necessary for deeper injuries [11,12].

Curiosa^®^ gel’s (Richter Gedeon Nyrt., Budapest, Hungary) main component is zinc-hyaluronan, which promotes cell regeneration and has antioxidant effects, therefore, it contributes to faster wound closure. Moreover, zinc has antimicrobial properties and is a vital part of more than 1000 transcription factors and 300 enzymes from every class. Hyaluronan is an essential component in the extracellular matrix, and is able to modify cellular functions, through its hyaluronan receptors and viscoelastic attributes. The gel formulation helps in the application of the treatment while it creates a moist environment, thereby stimulating wound healing. Zinc-hyaluronan gel also relieves adverse effects from the injuries, such as wound pain and tissue inflammation [13].

This trial aimed to assess the effectiveness of a zinc-hyaluronan-containing gel since, in previous studies, rapid reepithelialization and low infection rates have been reported when applying this intervention in adults [14] and as a part of pediatric combined burn treatments [15]. However, its efficacy has not been investigated in pediatric facial thermal injuries according to our literature search.

## 2. Materials and Methods

### 2.1. Design

A single-center, non-comparative cohort study was conducted at the Surgical Division, Department of Pediatrics, Medical School, University of Pécs, Pécs, Hungary. Patient data were collected retrospectively from our database, from 1 January 2016 to 15 October 2021.

The clinical application of zinc-hyaluronan gel was accepted and permitted by our medical board of the Hungarian Pediatric Surgery Committee in 2010. Written informed consent to participate and to publish was obtained from the patients’ legal guardians.

### 2.2. Participants

During the study period, 66 children visited our clinic with facial burns. Inclusion criteria were being younger than 18 years old and suffering from a II/1 degree facial burn. Moreover, the children had to be treated in the first 72 h after the injury with zinc-hyaluronan gel, with no other intervention used until the scab’s separation from the wound bed—including skin grafting. Furthermore, inclusion required the absence of acute or chronic comorbidity; a burned total body surface area (TBSA) smaller than 10%; and the attendance of the patient on every follow-up examination.

Children were excluded from the study if they had only I-degree burns (10 patients), were treated with other interventions (13 patients), had more extensive burns than 10% TBSA (4 patients), received skin grafts (2 patients), had severe comorbidities (2 patients) or missed the follow-up (12 patients). The relatively high rate of patients not appearing on control examinations was associated with the COVID-19 pandemic.

### 2.3. Treatment Protocol

**Figure 1:**
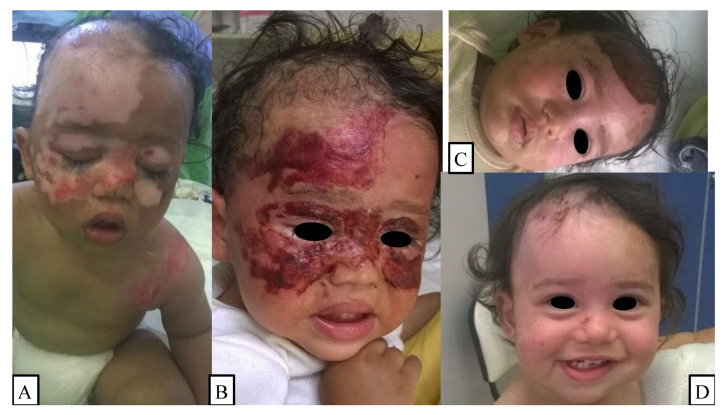
(**A**) A child had bullectomy and debridement performed in general anesthesia on the first day of injury. (**B**,**C**) On the third- and sixth-day control appointment, the patient showed signs of skin regeneration and reduction in wound size, which was covered with the biological membrane. (**D**) The 12-day follow-up revealed almost complete reepithelialization.

The children were transported to our hospital by their guardians or by ambulance after primary care. First, the attending surgeon assessed the patient’s medical history, as well as the wound depth and area according to the Lund–Browder diagrams for the appropriate age groups in addition to looking for signs of inhalation injury and eye trauma [16]. Clinical management began with the rinsing of the burn injury using soap and water, which was followed by disinfection of the affected area. Then, bullectomy and debridement of the necrotic tissue were performed under the effects of anxiolytics and local analgesia or general anesthesia, when it was deemed necessary based on the patients’ wound or age (Figure 1A). Next, the zinc-hyaluronan gel was applied to the cleaned burn surface, which was repeated 3–5 times daily until complete reepithelialization. It is important to note that this gel creates a biological membrane on top of the injury, which protects the area from water loss and infections, but it also makes the precise healing assessment difficult until complete wound closure (Figure 1B,C). Subsequent to the scabs’ separation from the wound bed, we advised the patients to apply an oily ointment, e.g., Vaseline, on their scars during the daytime, 3–4 times every day (Figure 1D). After showering, the usage of a heparin-sodium, allantoin, and extractum cepae-containing gel (Contractubex^®^ gel, Merz Pharmaceuticals GmbH, Frankfurt, Germany) was advocated due to its loosening and smoothing effect on sprawling or shrunken scar tissue, while reducing the itching sensation and inflammation [17]. Follow-ups were held on the day after the injury, and every 3–5 days thereafter, until wound closure. The children’s facial development and the scar tissue were reexamined 1, 6, and 12 months following the accident.

### 2.4. Outcomes and Demographics Measured

Each patient’s burn status was documented and photographed before applying the first dressing and thereafter at every control appointment until complete wound closure. The children were evaluated based on nine aspects. We analyzed the patients’ demographic data, such as sex and age distribution, the mechanisms and depth of the burns, and the injured TBSA, along with the associated burn regions. The primary outcomes were the average days until the occurrence of a complete, shiny, new layer of epithelium, the total time to reepithelialization (TTRE), the length of hospital stay (LOS), and the complication rates.

### 2.5. Statistical Analysis

The data on the outcomes were statistically analyzed in Microsoft Excel 2021, while for data presentation Microsoft PowerPoint 2021 (Microsoft Corporation, Redmond, Washington, USA) was used. Means and standard deviations (SDs) were calculated for the evaluated endpoints.

## 3. Results

**Figure 2:**
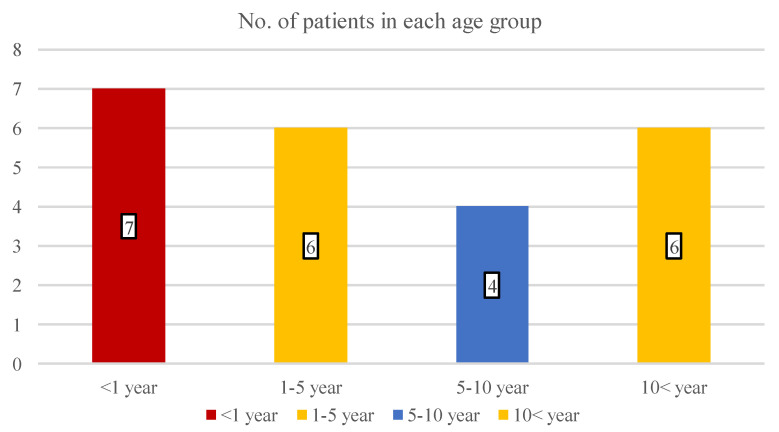
The number of children in four different age groups.

During the trial period, 23 children were eligible consecutively to participate in this cohort study. A total of 16 boys (69.6%) and 7 girls (31.4%) had II/1-degree facial burn with an average age of 6.2 years (SD: 5.8; range: 1–17 years). The patients’ distribution in the age groups is shown in Figure 2, which draws attention to the increased incidence in the infant population (30.4% of all patients).

**Figure 3:**
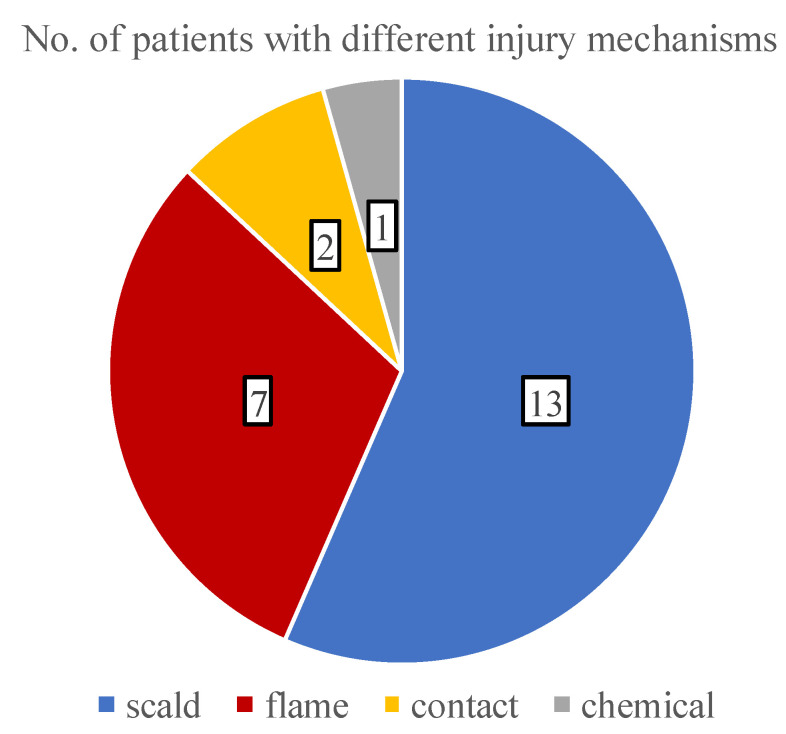
A pie chart showing the distribution of various burn etiologies.

With the analysis of the burn etiologies, we found that all of the traumas were accidental and the most common mechanism was scalding (56.5%), followed by flame (30.4%), contact (8.7%), and chemical injuries (4.3%) (Figure 3). During infancy, the incidence of scalding was 85.7% (6 out of 7 patients), while in teenagers, the risk of flame-caused burns increased to 66.6%.

**Figure 4:**
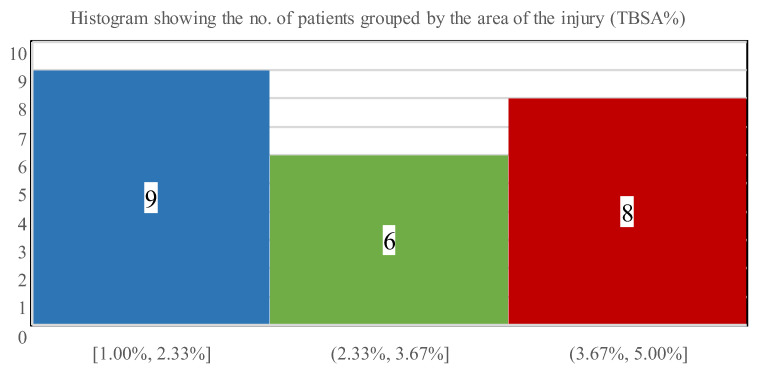
The number of patients stratified via affected burn surface ranges.

Four children had predominantly I-degree burns, while three patients had II/2-degree thermal injuries, in addition to their II/1-degree burns. Figure 4 shows the distribution of the burned facial areas with a mean of 3% TBSA (SD: 1.0; range: 1–5% TBSA).

**Figure 5:**
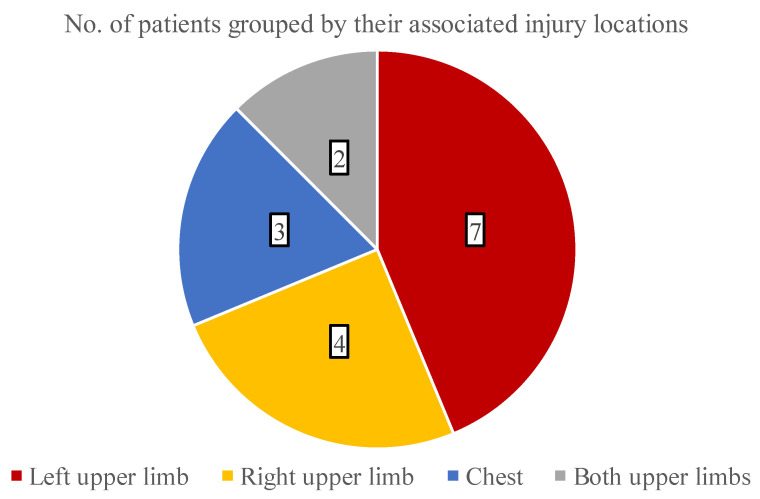
The distribution of burned areas associated with facial combustions.

The analysis of additional thermal injuries besides facial burns was also conducted, which is presented in Figure 5. Overall, 11 children suffered burns in multiple areas and most commonly (30.4% of all cases) the left upper limb was damaged.

**Figure 6:**
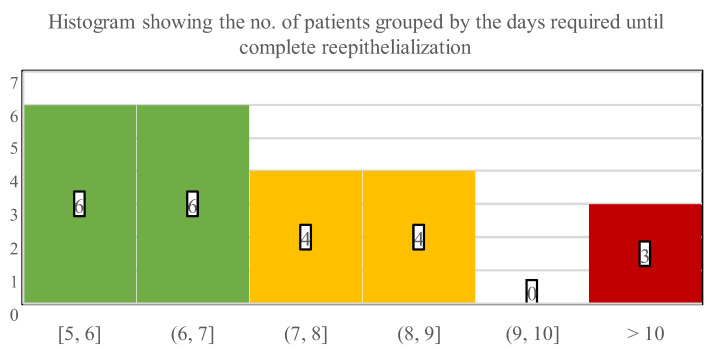
Numbers below the histogram indicate the time interval in days that was necessary for wound closure, while numbers in the bars indicate the number of patients who healed in the corresponding interims.

Our main evaluated endpoint was the days required for complete wound closure or TTRE of zinc-hyaluronan treated facial burns. On average, the children needed 7.9 days until reepithelialization (SD: 2.3; range: 5–15 days). Most patients (52.2%) healed within 5 to 7 days, while 34.4% of the children required 8–10 days (Figure 6). TTRE was more than 10 days in the remaining 13% of the population, who suffered 5 TBSA% injuries in the facial region and had associated burn locations as well.

**Figure 7:**
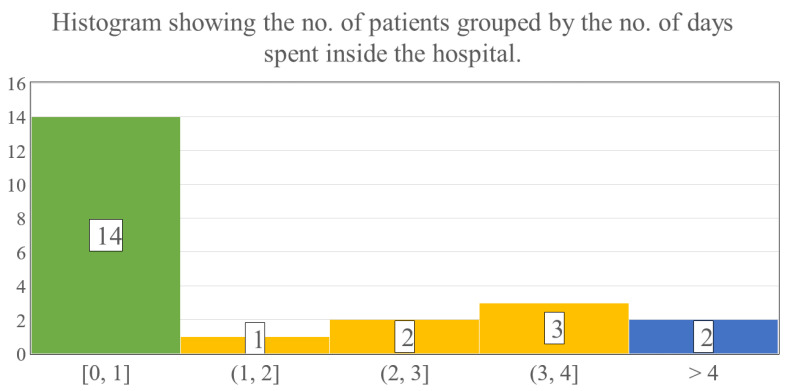
The numbers in the bars show the number of patients, while the numbers below the histogram indicate the time intervals in days spent hospitalized.

The evaluation of LOS revealed that 11 children could be treated ambulatory, thus the mean overall hospitalization was notably low, on average 2.0 days (SD: 3.8; range: 0–14 days) (Figure 7). When only the inpatient’s LOS was estimated, it showed that children who were admitted to our ward required 3.8 days (SD: 3.2; range: 1–14 days) until they could be discharged.

Lastly, complication rates were also assessed. Only two children had a fever, which peaked at 38 °C and 40 °C, even though all the wound cultures showed normal bacterial growth. Thus, these instances were attributed to noninfectious systemic inflammatory response syndrome (SIRS), which presumably developed due to the pro-inflammatory cytokine release in large area burn injuries [18]. Furthermore, none of the children showed signs of hypertrophic scarring or contractures, although some of the patients’ follow-up examinations are still ongoing.

## 4. Discussion

The qualities of zinc-hyaluronan gel make it an appropriate match for treating facial burns in children. Its ease of application and spontaneous separation, compared to traditional dressings, achieve wound healing in a painless and child-friendly manner [13]. Biological membranes formed over the injury act as a physical barrier against infection and fluid loss, while also promoting autolytic debridement and reepithelialization by creating a moist environment [14,19]. As a result of the facial region’s excellent vascularization, it allows for a more conservative approach even in deeper burns, compared to other parts of the body [20].

Pediatric facial burns still constitute a great challenge to healthcare providers, despite the availability of numerous treatment methods. Their burden is attributable to the presence of essential organs responsible for sensory, breathing, digestive, and communicative functions in this area. In this cohort study, we analyzed the demographic data and outcomes of 23 children whose facial II/1-degree thermal injuries were treated with zinc-hyaluronan gel, during a six-year period. Primarily boys were injured (69.4% of all patients) and the mean age of the patients was 6.2 years. A substantially increased incidence was observed in children who were younger than one year of age (30.4% of all cases). Burn etiology evaluation concluded that the patients were most commonly injured via scalding (56.6% of all children), which was even more predominant during infancy (85.7% of infants), while teenagers were more likely to be injured by flames (66.6% of teenagers). These results further validate the epidemiological observations provided by international trials [21,22,23].

The average wound area was 3% TBSA in the facial region, and the left upper limb was often burned concomitantly (30.4% of all cases). These thermal injuries epithelialized in 7.9 days on average, and the majority of the patients (52.2%) healed within 5 to 7 days when treated with the zinc-hyaluronan gel. It is critical to highlight that a significant percentage of the children (47.9%) could be treated in an ambulatory manner, thus the actual TTRE might be even lower because the patients’ wounds could not be examined each day. None of the children acquired infections and the regenerated skins were an almost perfect color match without any scarring. Moreover, due to the aforementioned reasons, the overall LOS was markedly low with 2.0 days on average. Because of the small TTRE and LOS values combined with the absence of complications, patients could recuperate at home. These factors could be also associated with a reduced total cost of care, shorter time required for dressing changes, and a lower number of anesthesias administered, in addition to decreased need for hospital staff and operating theatre availability. Regrettably, information was not collected about these aspects in a rigorous manner, but further research may prove them superior compared to other conventional approaches.

Research on pediatric facial burn interventions is scarce and there is no internationally accepted “gold standard”. Therefore, the choice of treatment mainly depends on the preference of the burn specialist and the customs of the healthcare institute. Similar depth and area injuries in children showed comparable (Mepitel, TBSA: 3.29%, TTRE: 7.58 days) [24] or delayed reepithelialization—related to zinc-hyaluronan—while using EZDerm (TBSA: 4.26%, TTRE: 18.75 days) [25,26], Acticoat (TBSA: 3.23%, TTRE: 14.18 days) [27,28], and Mepilex (TBSA: 2.85%, TTRE: 10.29 days) [26,27] according to the results of our previous meta-analysis [29]. It must be mentioned that these studies did not specifically evaluate facial burns and their application may be difficult in this area. Anesthesia could be necessary during dressing changes, thus a prolonged LOS was also observed with these treatments (EZDerm: 3.36 days, Mepilex: 3.12 days) [26]. The infection rate was also high in the last two interventions (EZDerm: 36.96%, Mepilex: 16.39%) [25,26,27], while there was no infection observed with Mepitel [24].

As reported by Rogers et al. [30], Biobrane may increase reepithelialization further (TBSA: 6%, TTRE: 10 days), although this alternative also raised the risk for infection (5.7%) and allergic reactions, while the overall LOS was also higher by 3.5 days on average. Other temporary dressings such as amnion membrane allografts (TBSA: 7.39%, TTRE: 13.33 days) [31] and tilapia xenografts (TBSA: 11.13%, TTRE: 10.07 days) [32] showed great healing potential as well, though their acquisition and storage could pose difficulties.

Limitations of this study must also be discussed. The relatively low sample size and single-center design might put into question the generalization of the results. Patients and healthcare providers could not be blinded; thus, the measurements might contain biases. Large population randomized controlled trials (RCTs) would be needed to conclude the efficacy of zinc-hyaluronan on pediatric facial II/1-degree burns. Yet, the findings in this study can be beneficial to outline the objectives and methods for future RCTs.

## 5. Conclusions

In summary, treating paediatric facial superficial partial-thickness burns with zinc-hyaluronan gel resulted in rapid wound closure and low complication rates, which were accountable for the low amount of hospitalizations. These advantages, in addition to the minimal need for anesthesia, and the gel’s ease of applicability and spontaneous separation, are associated with child-friendly burn care.

## Data Availability

Data is contained within the article.
